# A nanoparticle vaccine displaying the ookinete PSOP25 antigen elicits transmission-blocking antibody response against *Plasmodium berghei*

**DOI:** 10.1186/s13071-023-06020-8

**Published:** 2023-11-06

**Authors:** Guixiang Yao, Hui Min, Xinxin Yu, Fei Liu, Liwang Cui, Yaming Cao

**Affiliations:** 1 Department of Immunology, College of Basic Medical Sciences, China Medical University, Shenyang, Liaoning China; 2 Department of Internal Medicine, Morsani College of Medicine, University of South Florida, 3720 Spectrum Boulevard, Tampa, FL USA

**Keywords:** Malaria, Vaccines, Virus-like particles, SpyTag/SpyCatcher, Transmission-blocking activity

## Abstract

**Background:**

Safe and effective vaccines are crucial for the control and eventual elimination of malaria. Novel approaches to optimize and improve vaccine efficacy are urgently required. Nanoparticle-based delivery platforms are considered potent and powerful tools for vaccine development.

**Methods:**

In this study, we developed a transmission-blocking vaccine against malaria by conjugating the ookinete surface antigen PSOP25 to the *Acinetobacter phage* coat protein AP205, forming virus-like particles (VLPs) using the SpyTag/SpyCatcher adaptor system. The combination of AP205-2*SpyTag with PSOP25-SpyCatcher resulted in the formation of AP205-PSOP25 complexes (VLP-PSOP25). The antibody titers and avidity of serum from each immunization group were assessed by ELISA. Western blot and IFA were performed to confirm the specific reactivity of the elicit antisera to the native PSOP25 in *Plasmodium berghei* ookinetes. Both in vitro and in vivo assays were conducted to evaluate the transmission-blocking activity of VLP-PSOP25 vaccine.

**Results:**

Immunization of mice with VLP-PSOP25 could induced higher levels of high-affinity antibodies than the recombinant PSOP25 (rPSOP25) alone or mixtures of untagged AP205 and rPSOP25 but was comparable to rPSOP25 formulated with alum. Additionally, the VLP-PSOP25 vaccine enhanced Th1-type immune response with remarkably increased levels of IgG2a subclass. The antiserum generated by VLP-PSOP25 specifically recognizes the native PSOP25 antigen in *P. berghei* ookinetes. Importantly, antisera generated by inoculation with the VLP-PSOP25 could inhibit ookinete development in vitro and reduce the prevalence of infected mosquitoes or oocyst intensity in direct mosquito feeding assays.

**Conclusions:**

Antisera elicited by immunization with the VLP-PSOP25 vaccine confer moderate transmission-reducing activity and transmission-blocking activity. Our results support the utilization of the AP205-SpyTag/SpyCatcher platform for next-generation TBVs development.

**Graphical abstract:**

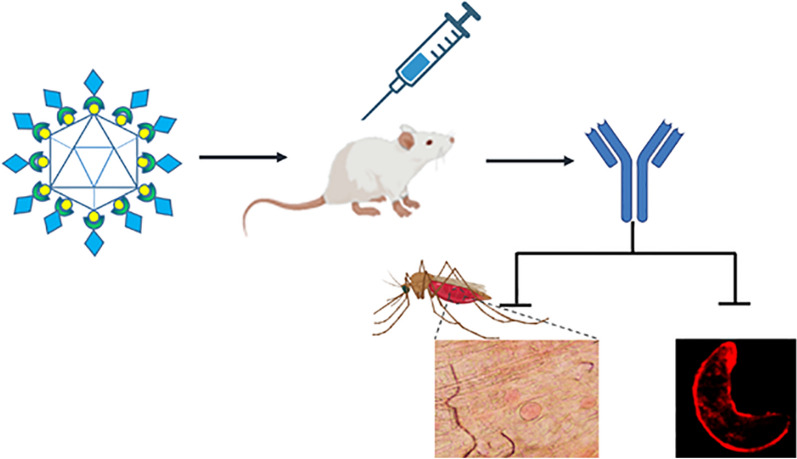

**Supplementary Information:**

The online version contains supplementary material available at 10.1186/s13071-023-06020-8.

## Background

Malaria is a life-threatening disease caused by infection with the *Plasmodium* parasites, transmitted through the bite of infected female *Anopheles* mosquitoes [1]. According to the World Malaria Report, there were approximately 627,000 deaths from malaria in 2020 [2]. This surge is mainly related to the global pandemic caused by COVID-19. Due to the increased resistance of *Plasmodium* parasites to anti-malarial drugs and the spread of insecticide-resistant mosquitoes [3–5], a highly effective transmission-blocking vaccine (TBV) would be a promising supplemental tool to control or even eliminate malaria. The principle of this vaccine is that functional antibodies against target antigens include proteins expressed on the surface of gametocytes/gametes/zygotes/ookinetes prevent the sexual reproduction of parasites in mosquito vectors when fed with gametocyte-infected blood [6]. The ookinete development and crossing of the mosquito midgut epithelium are significant bottlenecks in the life cycle of *Plasmodium* [7] and are prime targets for transmission-blocking strategies [8]. One of the leading TBV candidate antigens expressed during these critical phases is putative secreted ookinete protein 25 (PSOP25), a highly conserved ookinete surface protein, which is necessary for ookinete maturation in *Plasmodium berghei* [9, 10]. However, the transmission-blocking activity (TBA) of antibodies against this bacterially expressed recombinant protein is only modest. Therefore, developing a new platform to enhance its efficacy is imperative.

Among the novel strategies for vaccine development, virus-like particles (VLPs) or nanoparticle-based delivery platforms are considered safe and powerful tools that can enhance vaccine immunogenicity and efficacy [11]. VLPs are multiprotein structures with similar size and conformation to the respective native viruses. They can therefore be drained directly into lymph nodes and uptake effectively by antigen-presenting cells [12–14]. Moreover, their repetitive and dense surface structure allows for complement fixation and potent B cell receptor clustering, promoting B cell activation and resulting in stronger humoral and cellular immune responses [15, 16]. Importantly, as VLPs do not contain the original viral genetic material, they cannot replicate, making them non-infectious and highly safe [17]. Several VLP-based vaccines have been approved for clinical use, including vaccines against the hepatitis B virus, hepatitis E virus, and human papillomavirus [18], further demonstrating the medical application potential of the VLP technology. Various techniques, such as genetic fusion [19], chemical cross-linking [20], and split-protein (SpyTag/SpyCatcher) conjugation (plug-and-display) [21–23], are available to display multi-epitope antigens on the surface of VLPs. The former two methods, however, may prevent the expression or self-assembly of the particles and hamper the display of epitopes when the inserted antigen fragment is large [24, 25]. The latter approach involves the conjugation of antigens to self-assembled VLPs through isopeptide bonds that spontaneously form between the SpyTag peptides and SpyCatcher proteins, enabling heterologous antigen expression and VLP assembly [26, 27].

In this study, we used the AP205-SpyTag/SpyCatcher conjugation system to display PSOP25 on the surface of preassembled bacteriophage AP205 virus-like particles. Specifically, a genetically modified AP205 VLP presenting two SpyTag peptides per VLP subunit (AP205-2*SpyTag) was engineered, while the PSOP25 antigen was genetically fused to the SpyCatcher at C-terminus (PSOP25-SpyCatcher). VLP display of PSOP25 antigens occurred when AP205-2*SpyTag was mixed with PSOP25-SpyCatcher. The immunogenicity of the PSOP25-based VLP vaccine itself was assessed without the addition of exogenous adjuvants in BALB/c mice.

## Methods

### Experimental mice and parasites

Female BALB/c mice 6–8 weeks of age were purchased from the Beijing Animal Institute (Beijing, China) and maintained at China Medical University central animal facilities. The *P. berghei* ANKA 2.34 strain was maintained by serial passages and used for challenge infections or ookinete culture. All animal experiments were conducted in accordance with the protocols approved by the Animal Ethics Committee at China Medical University.

### Design, expression, and purification of AP205-2*SpyTag

The *Acinetobacter phage* AP205 coat protein (Gene ID: 956335) was modified to create the AP205-2*SpyTag, which displays two SpyTags per VLP subunit. Briefly, the SpyTag peptide sequence (AHIVMVDAYKPTK) was added to both the N- and C-termini of the AP205 coat protein with a flexible linker containing GSGTAGGGSGS at the N-terminus and GTASGGSGGSG at the C-terminus of AP205-2*SpyTag [26]. The gene sequence was inserted into the pET-20b ( +) vector (Novagen) using *Bam*HI and *Not*I (New England Biolabs) restriction sites, and underwent codon-optimization for bacterial recombinant expression prior to synthesis (Genscript). The AP205-2*SpyTag was expressed in *E. coli* Rosetta-gami B (DE3) cells under induction with 1 mM IPTG (Sigma) at 20 °C for 16 h. Bacterial cells from the overnight culture were pelleted by centrifugation at 4 °C. The pellet was resuspended with a HIS-binding buffer containing 50 mM sodium phosphate buffer and 300 mM NaCl (pH 8.0) and was lysed by sonication [28]. The bacterial lysate was centrifuged, and the pellet was suspended in Buffer B solution (100 mM NaH_2_PO_4,_ 10 mM Tris, 8 M Urea, pH 8.0). After centrifugation, the supernatant was then filtered through 0.22 μm filters and incubated with Ni–NTA His-Bind Superflow resin (Novagen) at room temperature (RT) for 1 h. Nickel-captured AP205-2*SpyTag was then washed three times in Buffer C solution (100 mM NaH_2_PO_4,_ 10 mM Tris, 8 M urea, pH 6.3) to remove impurity protein. The protein was eluted into 5-ml fractions with Buffer E solution (100 mM NaH2PO4, 10 mM Tris, 8 M urea, pH 4.5) and dialyzed in buffers with decreasing urea concentration. The preparation was subjected to 10% SDS-PAGE analysis, and the desired fractions were concentrated to a range of 1000–2000 μg/ml. In parallel, the untagged AP205 in control groups was expressed and purified from inclusion bodies.

### Design, expression, and purification of PSOP25-SpyCatcher

The gene fragment of PSOP25 (PBANKA_1119200) corresponding to amino acids 45–245 of the predicted protein sequences [10] was fused with a 6 × histidine purification tag (HIS) at the N-terminus and the SpyCatcher (24–139 aa) at the C-terminus. A flexible GGSGS linker was inserted between the PSOP25 and the SpyCatcher. The final construct was codon-optimized for recombinant expression in *Escherichia coli* and synthesized by Genscript. The PSOP25-SpyCatcher expression sequences were cloned into the vector pET-32a ( +) (Novagen) at the *Bam*HI and *Not*I sites and transformed into the BL21 (DE3) *E. coli* strain. The recombinant protein was expressed under the induction with 0.5 mM IPTG at 19 °C for 12 h. For the purification of soluble proteins, bacterial cultures were harvested and lysed using the same protocol as for inclusion bodies. The resulting supernatant was filtered and incubated with Ni–NTA His-Bind Superflow resin. The resin was then washed four times at 30 mM imidazole and eluted with 250 mM imidazole. Purified PSOP25-SpyCatcher was desalted through dialysis in 0.1 M PBS (pH 7.4) at 4 °C overnight. The purity of the recombinant protein was assessed by 10% SDS-PAGE. The purification of rPSOP25 protein in control groups also followed this procedure.

### Conjugation of PSOP25 to VLPs

To couple PSOP25 with VLPs, purified AP205-2*SpyTag protein was mixed with the PSOP25-SpyCatcher protein at a molar ratio of 1:3 in standard PBS supplemented with 0.2% Polysorbate 80 (pH 7.2) and incubated overnight at 4 °C. The conjugated sample was analyzed using Coomassie-stained SDS-PAGE to determine the number of VLP monomer subunits that had been coupled to PSOP25 via SpyTag/SpyCatcher interaction.

### Transmission electron microscopy (TEM)

The formation of VLP particles was determined by TEM after negative staining. Briefly, 10 μl VLP-PSOP25 was diluted to a concentration of 0.2 mg/ml in H_2_O and applied to 200 mesh copper grids coated with a thin carbon film for 15 min. The sample was blotted with filter paper, stained in the dark with 2% phosphotungstic acid for 15 s, then blotted and air dried at RT. Grids were imaged using a Hitachi HT-7700 transmission electron microscope at an accelerating voltage of 80 kV.

### Mouse immunization procedure

To investigate the immunogenicity of the conjugated proteins, mice were immunized with the following protocol: age-matched BALB/c mice (*n* = 5 for each group) were immunized subcutaneously with 200 µl of the vaccine design. This corresponds to a dose of 50 μg PSOP25 per mouse, either as a monomer or conjugated as a VLP-PSOP25 particle, while the PBS control group was injected with PBS only. The immunized mice received two booster vaccinations of 25 μg PSOP25 antigen each at weeks 2 and 4 following the primary injection. Blood samples were collected on days 14, 28, and 38. Immune sera were isolated from whole blood by allowing samples to clot at RT for 2 h before centrifugation at 12,000 ×*g* for 15 min. Pooled antisera were then extracted and stored for subsequent trials.

### Serum IgG, IgG1, and IgG2a levels

Enzyme-linked immunosorbent assay (ELISA) was performed on days 14, 28, and 38 after the first vaccination to analyze the antibody response, including anti-PSOP25 IgG levels, titers, and PSOP25-specific serum IgG1 and IgG2a levels. Purified rPSOP25 protein was prepared at a concentration of 10 μg/ml in 0.05 M sodium carbonate buffer (pH 9.6), followed by overnight incubation at 4 °C in each well of a 96-well ELISA plate. After washing three times with PBS/T (0.05% Tween 20 in PBS), the plates were blocked at 37 °C for 1 h with 1% bovine serum albumin (BSA, Sigma) in PBS and then washed in PBS/T as before. For IgG quantification, mouse serum samples were diluted 1:200 in a blocking buffer. For antibody titration, pooled mouse antisera were serially diluted with 1% BSA in PBS from 1:2000 to 1:256,000. For IgG isotype analysis, mouse serum pooled from different immunization groups was diluted 1:50 in PBS supplemented with 1% BSA. Subsequently, each well was loaded with 100 μl of the respective dilution and incubated at 37 °C for 2 h. After four washes with PBS/T, HRP-conjugated goat anti-mouse IgG (Invitrogen, USA) diluted 1:10,000 in PBS was incubated at 37 °C for 1 h to measure IgG levels and antibody titers. For antibody subtype detection, HRP-labeled goat anti-mouse IgG1 or IgG2a at a 1:5000 dilution was applied to appropriate wells and incubated for 1 h at 37 °C. After the last four washes in PBS/T, 100 μl of TMB substrate solution was added to each well and incubated at RT for 10 min in a dark place. Finally, color reactions were terminated by adding 100 μl of stop solution to each well, and the optical density (OD) was immediately determined at 450 nm using a microplate reader. The serum IgG endpoint titers were defined as the highest antiserum dilution at which the OD value at 450 nm exceeded the mean of PBS negative control + 3 × SEM.

### Avidity of serum IgG measured by ELISA

The avidity index of anti-PSOP25 antibodies was determined using the modified ELISA assay; this procedure is similar to the ELISA protocol used to measure antibody levels (described above), except that after the incubation of the plates with diluted serum, 100 μl 8 M urea was added to each well for 20 min (reference wells were treated with PBS). The avidity index values were calculated as the ratio of the mean ELISA absorbance value of 8 M urea-treated wells to control wells incubated in PBS multiplied by 100 [26]. In addition, all serum samples were diluted based on pre-determined OD450 values for normalization of IgG levels and tested in duplicate.

### Indirect immunofluorescence assay (IFA)

To confirm the recognition of native parasite PSOP25 antigen by VLP-PSOP25-induced antiserum, IFA was conducted on ookinetes that were obtained by mixing the gametocyte-infected blood with the ookinete culture medium [50 mg/l penicillin, 50 mg/l streptomycin, 100 mg/l neomycin, 25% (*v*/*v*) fetal bovine serum and 6 U/ml heparin in RPMI 1640, pH 7.5] and maintained at 19 °C for 24 h. The ookinete cultures were fixed with 4% paraformaldehyde and 0.0075% glutaraldehyde at RT for 30 min, followed by PBS washing. The fixed parasites were permeabilized with 0.1% Triton X-100 in PBS at RT for 10 min, then washed again in PBS and blocked with blocking buffer (3% BSA in PBS) at RT for 1 h. The cells were incubated with VLP-PSOP25-induced antisera (1:500), rPSOP25 + alum-induced antisera (positive control, 1:500), or sera obtained from PBS-immunized mice (negative control, 1:500), respectively. Additionally, all samples were co-incubated with rabbit antisera against Pbs25 (1:500) as a marker for ookinetes at 37 °C for 1 h and subsequently washed three times with PBS. After incubating with Alexa Fluor 488-conjugated goat anti-mouse IgG secondary antibodies (1:500, Invitrogen, USA) and Alexa Fluor 555-conjugated goat polyclonal antibody to rabbit IgG. (1:500, Abcam, UK) at RT for 1 h, the cell nuclei were counterstained with Hoechst 33,258 solution (1:1000; Sigma). All samples were imaged and analyzed using a fluorescence confocal laser scanning microscope (STELLARIS 5, Leica Microsystems).

### Purification of ookinetes and Western blot

To promote the culture and development of ookinetes, mice were treated with phenylhydrazine (Sigma-Aldrich) three days prior to intraperitoneally injection of 5 × 10^6^ *P. berghei* infected red blood cells (iRBCs). Once parasitemia reached approximately 10%, the cardiac puncture was performed on each mouse to collect 1 ml of blood, which was then added to 9 ml of ookinete medium and incubated at 19 ℃ for 24 h. A culture smear was taken to observe the state of ookinete growth. The ookinetes located at the gray interface were isolated and collected through gradient density centrifugation utilizing 62% (*v*/*v*) Nycodenz/PBS cushion. Following two washes with 0.01 M PBS, purified ookinetes underwent treatment with 0.02% saponin (Sigma) to lyse erythrocytes before being washed with PBS. The ookinete proteins were extracted using 2% SDS supplemented with 1 × protease inhibitors. Equal amounts of protein lysates (30 μg per lane) were boiled and separated on a 10% SDS-PAGE gel and electro-transferred to a PVDF membrane (Bio-Rad). The membrane was blocked at RT for 2 h using 5% skimmed milk in TBST, following incubated with mouse sera (1:200) from different immunized groups, i.e. polyclonal antisera raised from VLP-PSOP25, rPSOP25 + alum (positive control) or PBS (negative control) immunization. After four washes in TBST, membranes were incubated with HRP-conjugated goat anti-mouse secondary antibody (1:20,000, Invitrogen) at RT for 2 h. An ECL Western Blotting Kit was used to visualize the protein bands.

### Inhibition of ookinete formation assays

The functional activity of specific antisera was evaluated using two methods of ookinete formation inhibition assay, namely active immunization method and in vitro dilution method. The distinction between the two approaches lies in whether the immunized mice were directly infected by the parasites. For the active immunization approach, mice immunized with the respective vaccines were pretreated with phenylhydrazine and injected with 5 × 10^6^ *P. berghei* iRBCs 10 days after the third vaccination. Parasitemia was allowed to reach approximately 7–9% at 3 days post-infection (p.i.). When the mice had similar parasitemia and gametocytemia levels, tail blood samples of 10 μl per mouse were taken and mixed with 90 μl ookinete culture medium. The mixture was then maintained at 19 °C for 24 h. Then, 0.5 μl of mixed ookinete cultures were utilized to prepare smears, which were subsequently fixed and probed with anti-Pbs21 mAb (1:500) and Alexa Fluor 488-conjugated goat anti-mouse IgG (1:500). The cell nuclei were counterstained with DAPI. The number of ookinetes per well (equivalent to 0.5 μl of cultures) was determined by fluorescence microscope (Olympus, U-HGLGPS). For the in vitro dilution study, non-immunized mice were used as hosts for the parasites. For ookinete culture, antisera from different groups of immunized mice were diluted at 1:5 and 1:10 with each complete ookinete medium containing 10 μl of infected mouse blood in a total volume of 100 μl. Subsequently, the mixture was incubated at 19 °C for 24 h. Then, the number of ookinetes in 0.5 μl mixed cultures was determined using the same method as described in the active immunization protocol.

### Direct mosquito feeding assay

Phenylhydrazine pretreated BALB/c female mice were infected with 5 × 10^6^ *P. berghei* ANKA iRBCs. Three days after blood infection, parasitemia was evaluated, and male gamete exflagellation centers were quantified. Mice with similar parasitemia and gametocytemia were selected for mosquito feeding. Antisera (500 μl) collected from mice in different immunization groups were passively transferred into each selected infected mouse via the tail vein by slow injection. One hour following the serum transfer, 50 adult *Anopheles stephensi* mosquitoes pre-starved for 12 h were allowed to feed on each infected mouse for 2 h. The unfed mosquitoes were removed, and fully engorged mosquitoes were separated and maintained at 19 °C and 80% relative humidity. Nine days after the blood meal, 30 mosquitoes from each group were randomly selected for dissection. Their midguts were stained with 0.5% mercurochrome and examined under the microscope to count the number of oocysts per mosquito midgut [9]. TRA was expressed as the percent inhibition in oocyst intensity, while TBA was expressed as the percentage inhibition in the prevalence of infected mosquitoes [29].

### Statistical analysis

Statistical analysis of data was performed using GraphPad Prism 8.0 software. The antibody titers and number of ookinetes were analyzed by analysis of variance (ANOVA). The oocyst density was analyzed by the Mann-Whitney *U* test. Fisher’s exact test was employed to analyze the prevalence of infection. Statistical significance was defined as *P*-values < 0.05 in all cases.

## Results

### Generation of a VLP vaccine displaying PSOP25 using the SpyTag-SpyCatcher strategy

**Fig. 1:**
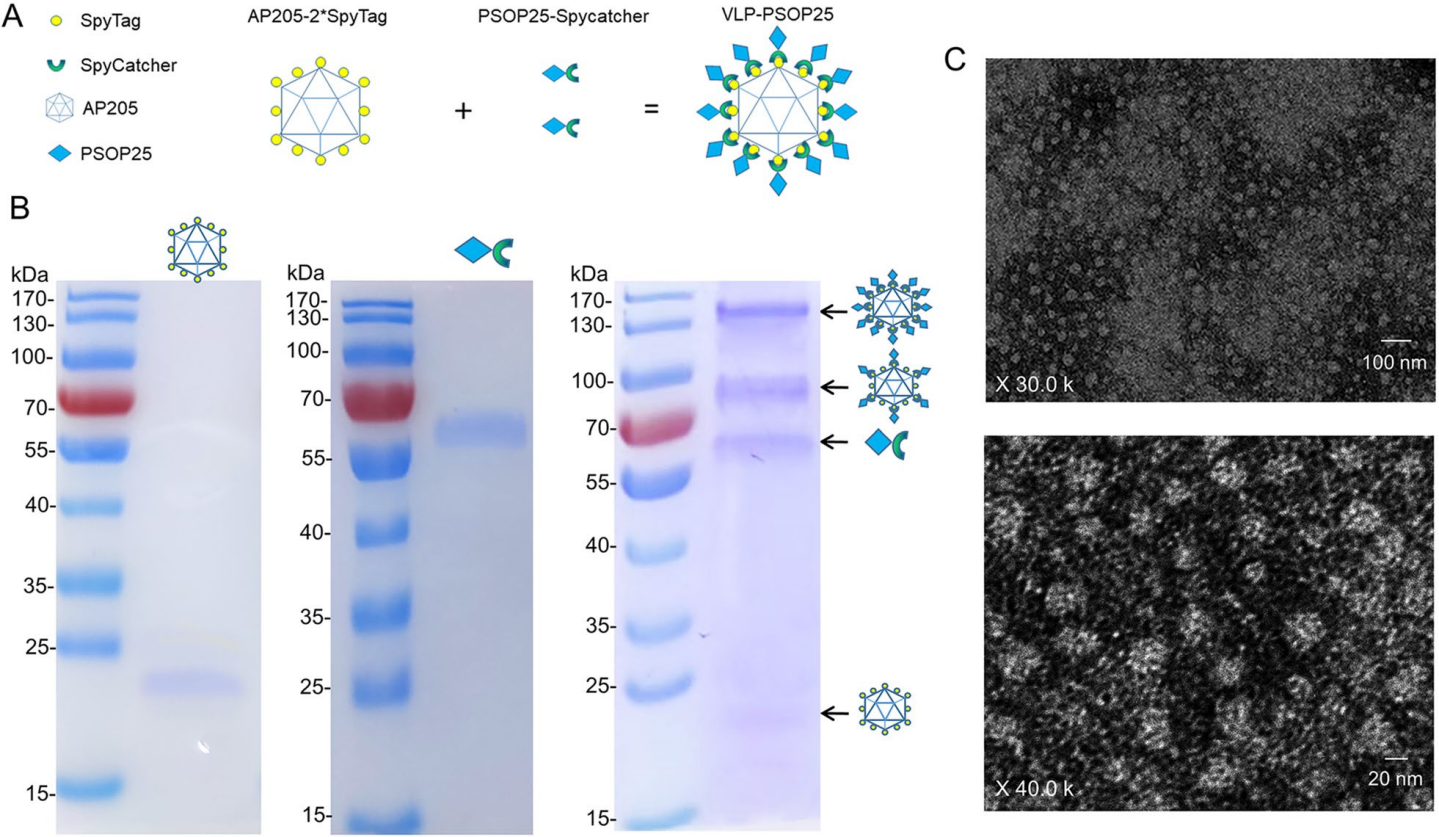
VLP-PSOP25 vaccine design and characterization. **a** Schematic diagram of AP205-2*SpyTag and PSOP25-SpyCatcher coupling via spontaneous isopeptide bonds formed between SpyTag and SpyCatcher. **b** Purified recombinant proteins/components were separated on 10% reduced SDS-PAGE gels and stained with Coomassie blue. The left panel shows the expected ~ 24 kDa band for purified recombinant AP205-2*SpyTag. The middle panel shows the purified recombinant PSOP25-SpyCatcher with a ~ 60-kDa band. The right panel demonstrates that mixing of AP205-2*SpyTag VLP with PSOP25-SpyCatcher resulted in four different bands from top to bottom representing as: a conjugate of two PSOP25 antigens bound to each end of an AP205-2*SpyTag protein (144 kDa), a conjugate of a single PSOP25 antigen and the AP205-2*SpyTag protein (84 kDa), uncoupled PSOP25 antigen (60 kDa), and unconjugated AP205-2*SpyTag protein (24 kDa). **c** Images of negative staining transmission electron microscopy for the VLP-PSOP25 vaccine. The top image shows uniform, non-aggregated particles of an average size of ~ 38 nm. The image below shows typical virus-like particle shapes

To construct the AP205 VLPs, the split-protein peptide SpyTag containing 13 amino acids was fused to both the N- and C-terminus (AP205-2*SpyTag) of the AP205 coat protein (Gene ID: 956,335). The split-protein SpyCatcher sequence containing 116 amino acids was fused to the C-terminus of PSOP25 (45–245 aa) to form PSOP25-SpyCatcher (Fig. 1a). We expressed AP205-2*SpyTag and PSOP25-SpyCatcher as His-tagged recombinant proteins in bacteria and purified the proteins using an affinity column. SDS-PAGE analysis showed that the purified recombinant proteins were relatively pure, with a molecular weight of approximately 24 and 60 kDa, respectively (Fig. 1b). Both protein bands reacted with the anti-His tag monoclonal antibody (Additional file 1: Fig. S1a, b). The yield of the recombinant proteins obtained from the bacterial expression system was approximately 1–2 mg/ml. In addition, the AP205 coat protein and rPSOP25 protein without the coupling tag were also expressed and purified for control purposes. SDS-PAGE and Western blot detected obvious target protein bands at 35 and 42 kDa, respectively (Additional file 2: Fig. S2 a–d), indicating that both proteins were successfully expressed and purified [10].

AP205-2*SpyTag and PSOP25-SpyCatcher were mixed at a molar ratio of 3:1 to allow the formation of PSOP25 VLPs, with the establishment of a covalent isopeptide bond between the SpyTag and SpyCatcher [26, 30] (Fig. 1a). Covalent coupling of the PSOP25 to the AP205 VLPs was confirmed by the detection of a protein band of 144 kDa with SDS-PAGE, corresponding to the added size of two PSOP25 antigens (60 kDa) and AP205 VLP subunit (24 kDa), as exemplified in Fig. 1b. Western blot analysis also detected the conjugated VLP-PSOP25 bands with anti-His monoclonal antibody (Additional file 1: Fig. S1c). Besides, negative-staining transmission electron microscopy (TEM) was used to reveal the presence of a homogeneous population of non-aggregated VLP-PSOP25 complexes with an average size of ~ 38 nm (Fig. 1c).

### Immunogenicity of VLP-PSOP25

**Fig. 2:**
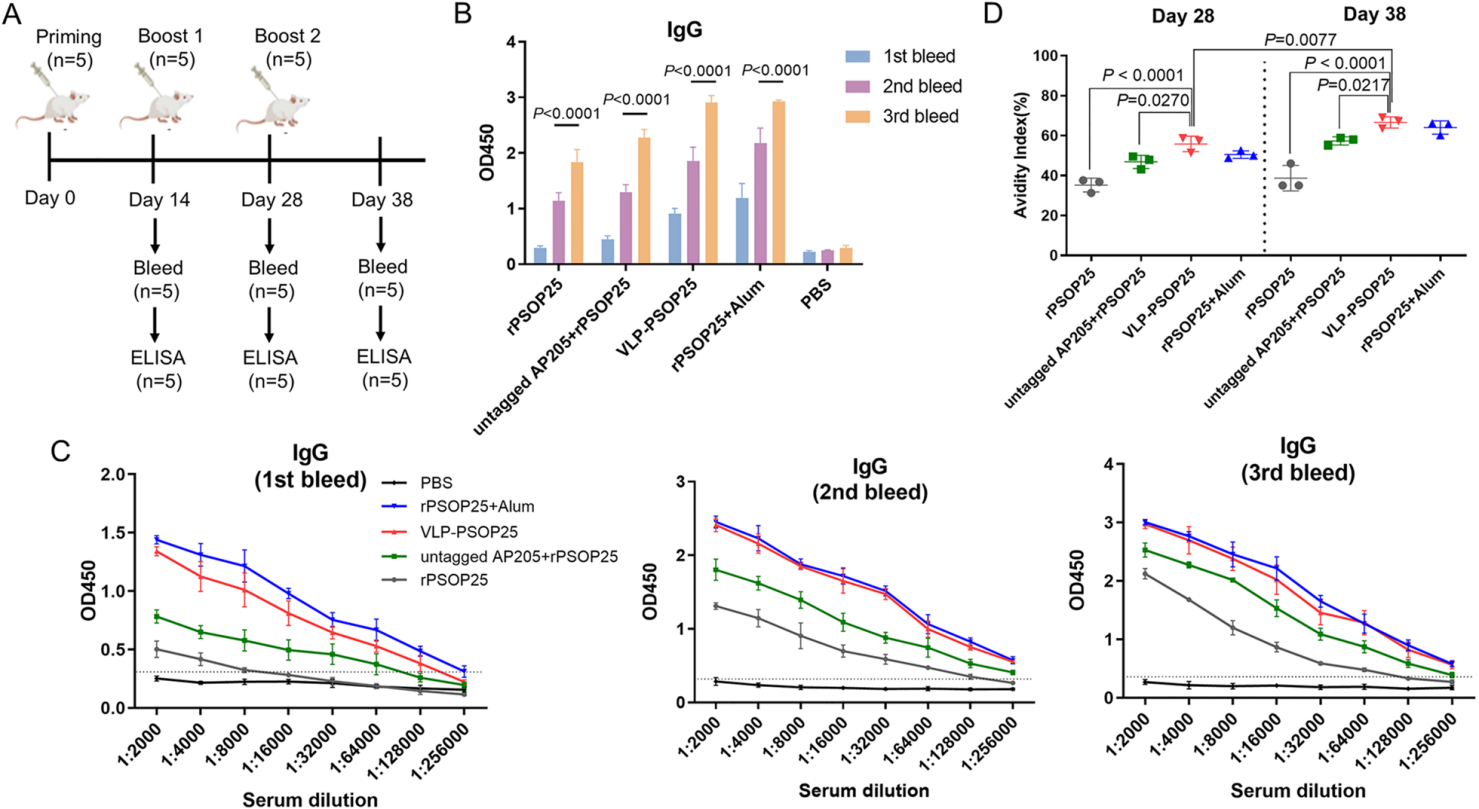
Antibody responses generated by each immunization group. **a** Schematic diagram of mouse immunizations and sampling. **b** PSOP25-specific IgG levels for three immunizations in the sera of immunized mice in each group. Sera were harvested and tested post-first immunization on days 14, 28, and 38. **c** PSOP25-specific IgG titers for three immunizations in the sera of immunized mice in each group. Serum samples were measured at two-fold serial dilution. The lower horizontal line (cut-off value) represents the average value of the negative control antisera + 3 × SEM. **d** The antibody avidity index of PSOP25-specific IgG was assessed on day 28 (left of dash line) and day 38 (right of dash line) with serum samples from mice in each group. Sera were treated with PBS or 8 M urea. The avidity index was calculated as the ratio of the ELISA OD490 value of 8 M urea-treated wells to control wells incubated in PBS. Error bars were indicated as mean ± SD

The immunogenicity of VLP-PSOP25 was assessed by the evaluation of vaccine-induced humoral responses in mice. It was compared with four control groups, including rPSOP25 antigen alone, untagged AP205 + rPSOP25, rPSOP25 + alum, and phosphate-buffered saline (PBS) group (Table 1). BALB/c mice were randomly divided into the above five immunization groups, with five mice in each group, and immunized subcutaneously on days 0, 14, and 28 with equivalent PSOP25 antigen (Fig. 2a). Antibody levels (Fig. 2b) or titers (Fig. 2c) were measured by ELISA with sera obtained on days 14, 28, and 38. The results showed that the third immunization elicited significantly higher levels of anti-PSOP25 IgG antibodies than the second immunization in all the groups containing the rPSOP25 antigen (*P* < 0.0001). The endpoint antibody titers were calculated as the highest serum dilution with an absorbance value exceeding the cutoff (defined as the mean of PBS negative control + 3 × SEM). Although antibody titers in mice after the first immunization immunized with VLP-PSOP25 (1:64,000) were lower than those from mice receiving rPSOP25 + alum (1:128,000) (*P* < 0.0001), there were no significant differences in antibody titers between these two groups after the second and third immunization (*P* > 0.05, Fig. 2c). Animals immunized with both VLP-PSOP25 and rPSOP25 + alum exhibited a robust immune response, as evidenced by anti-PSOP25 IgG antibody titer reaching 1:256,000 after booster administrations. These titers were higher than those observed in the untagged AP205 + rPSOP25 (1:128,000) and rPSOP25 (1:64,000) groups. Notably, at each time point tested, VLP-PSOP25 induced higher anti-PSOP25 IgG antibody titers compared to either rPSOP25-only or the untagged AP205 + rPSOP25 group (*P* < 0.01, ANOVA, Fig. 2c). This experiment confirmed that while rPSOP25 alone is poorly immunogenic, its immunogenicity could be enhanced through conjugation with VLPs or extrinsic adjuvants. These findings indicated that booster immunizations differentially increased the antibody titers of the VLP-PSOP25 vaccine, and VLP itself may provide a notable adjuvant effect.

**Table 1:** Groups of immunized mice and vaccine composition of different groups

Group (*n* = 5)	Immunogen	Classification
1	rPSOP25	Non-adjuvanted soluble protein control group
2	Untagged AP205 + rPSOP25	Unconjugated control group
3	VLP-PSOP25	Experimental group
4	rPSOP25 + alum	Positive control group
5	PBS	Negative control group

Antibody avidity is an essential parameter of the quality of vaccine-induced immune responses [31, 32]. The avidity of the serum IgG collected on days 28 and 38 was assessed by employing a chaotropic agent (urea) to disrupt the interactions between the antibody and the coating antigen in a modified ELISA assay [33]. The avidity index value of sera collected from the VLP-PSOP25 group after the third immunization (67%) was significantly higher than that after the second immunization (55.8%) (*P* = 0.0077). Furthermore, the antibody avidity from the VLP-PSOP25 group was significantly higher than from either the rPSOP25-only or untagged AP205 + rPSOP25 groups on days 28 and 38 (rPSOP25, *P* < 0.0001; untagged AP205 + rPSOP25, *P* < 0.05, ANOVA; Fig. 2d), although there was no significant difference compared with the rPSOP25 + alum group.

**Fig. 3:**
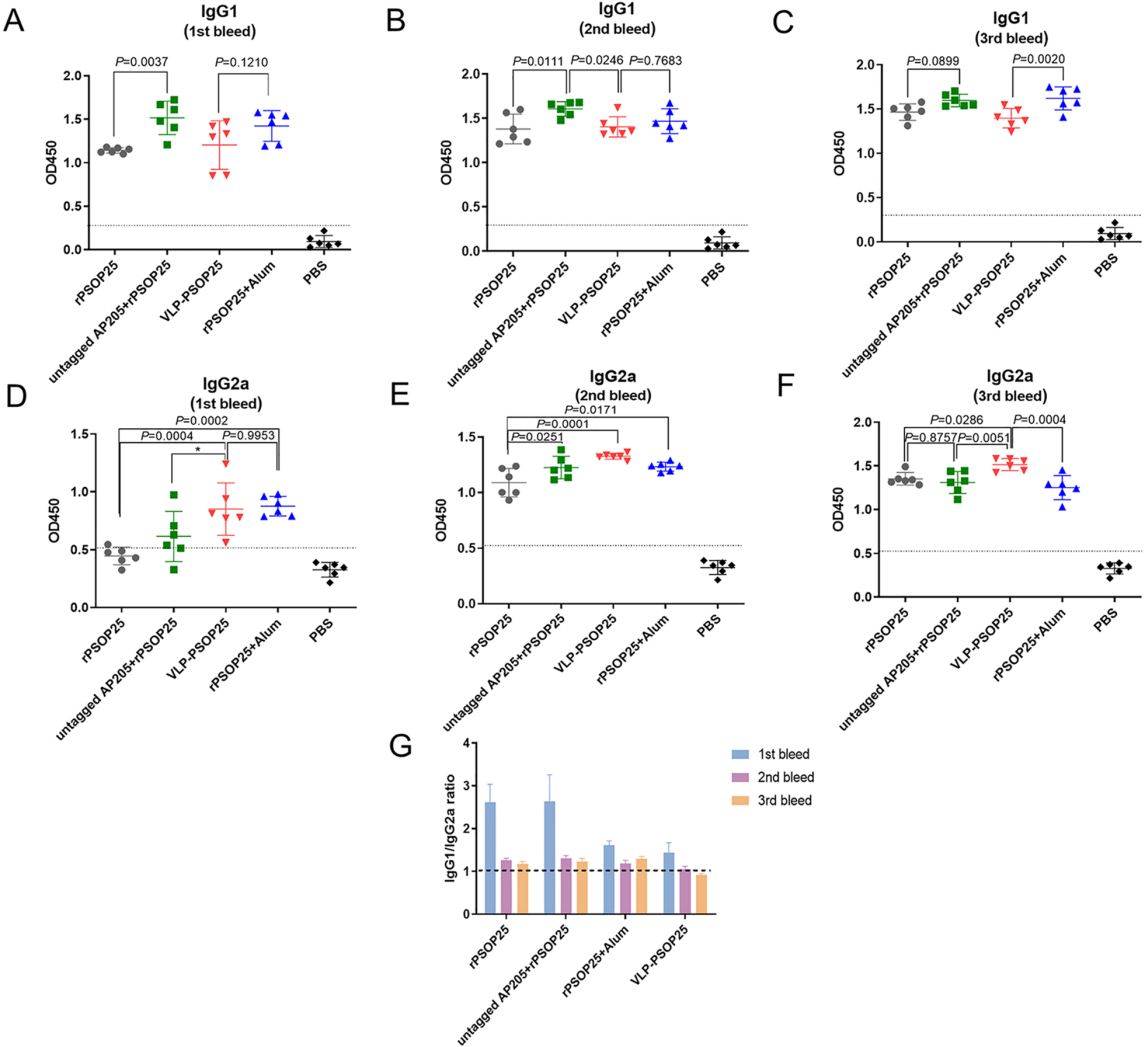
Analysis of antibody subclasses for three immunizations in the sera of immunized mice in each group. PSOP25-specific IgG1 (**a**–**c**) and IgG2a levels (**d**–**f**) were detected by ELISA at days 14, 28, and 38 after the first immunization, and the corresponding IgG1/IgG2a ratios are shown in the **g** panel. The data are representative of two experiments. In each panel (**a**–**f**), the lower horizontal line represents the mean of the negative control antisera + 3 × SEM. Error bars were representing mean ± SD

The induction of IgG antibody subclasses following vaccination can indicate the types of elicited immune responses. In mice, the production of IgG1 isotype is associated with a more engaged Th2-type response, while IgG2a is indicative of a Th1 bias. To elucidate the type of immune responses induced by the VLP-PSOP25 vaccine, PSOP25-specific IgG1 and IgG2a isotypes of antibodies were determined (Fig. 3a–f). Our analysis revealed that the rPSOP25 antigen alone, untagged AP205 + rPSOP25, and rPSOP25 + alum groups primarily elicit Th2-biased immune responses, as evidenced by the predominant induction of IgG1 antibodies at all time points. Interestingly, the VLP-PSOP25 immunized group showed a different tendency, with IgG1/IgG2a ratios of 1.05 and 0.91 on days 28 and 38, respectively, suggesting the induction of more balanced Th1 and Th2-type responses in mice (Fig. 3g). Compared to the rPSOP25 + alum group, VLP-PSOP25 induced a significantly lower level of IgG1 and a higher level of IgG2a in the final boost. The AP205 VLP platform appears to promote the production of IgG2a antibodies, consistent with previous findings [34, 35]. These results collectively demonstrate that the VLP-PSOP25 vaccine significantly enhances the immunogenicity of soluble PSOP25 antigen and elicits a balanced Th2/ Th1-type immune response.

### Confirmation of PSOP25-specific antibody responses

**Fig. 4:**
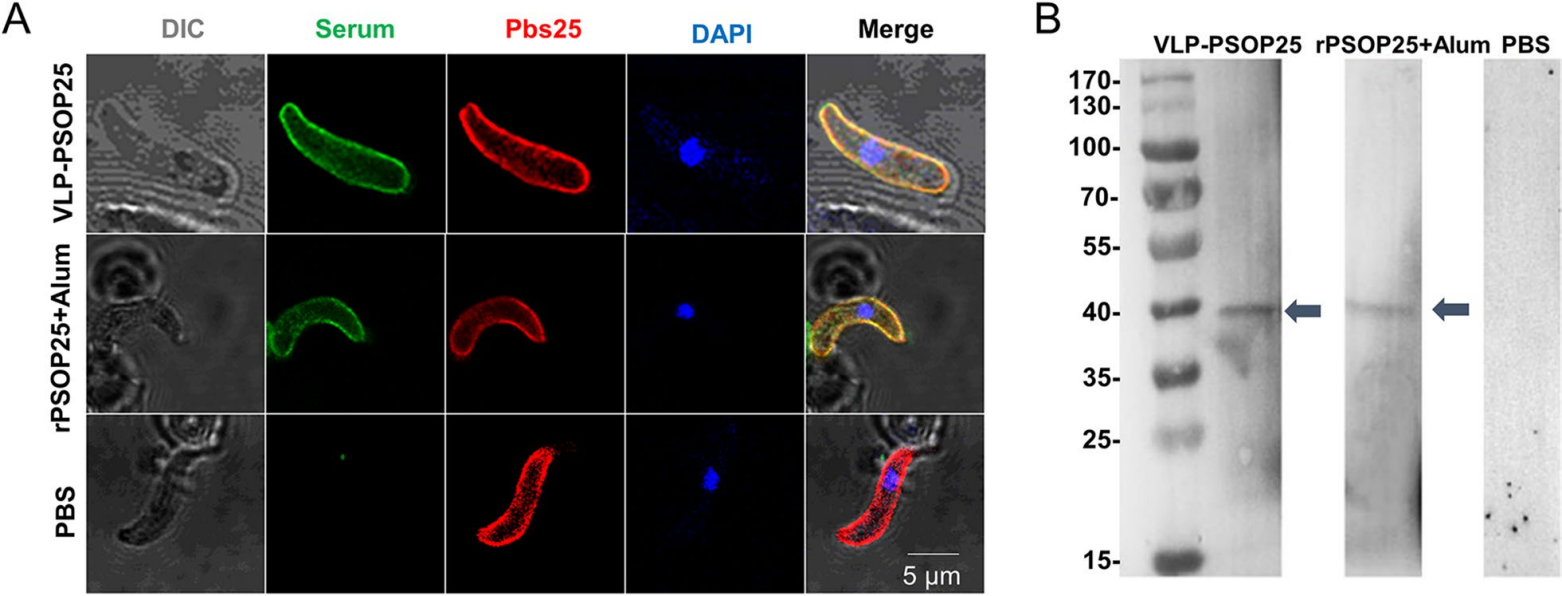
Reactivity of vaccine-induced antisera with the native PSOP25 antigen in ookinetes. **a** IFA was performed on ookinete using serum after VLP-PSOP25 (PolyAb). Positive control: serum after rPSOP25 + alum; negative control: a control mouse serum (PBS). All parasites were co-stained with antibodies against the marker for the ookinetes stage (Pbs25, red). Nuclei were stained with DAPI (blue). Scale bar = 5 μm. **b** Western blot analysis of purified *P. berghei* ookinete (Ook) lysates with serum after VLP-PSOP25 (PolyAb). The lysates at 30 μg per lane were subjected to electrophoresis under reducing conditions by 10% SDS-PAGE and probed with the corresponding antisera. Serum after rPSOP25 + alum was used as positive control; a control mouse serum (PBS) was used as a negative control. The arrows indicated the expected ~ 40-kDa band

PSOP25 is expressed on the membrane of the ookinetes [10]. We confirmed that the antisera targeting the rPSOP25 antigen recognized the native PSOP25 in *P. berghei* ookinetes using the immunofluorescence assays. Antisera against VLP-PSOP25 produced strong fluorescence signals on the ookinete surface, similar to the pattern with the rPSOP25 + alum-positive control serum (Fig. 4a). In contrast, serum from mice injected with PBS did not react with the native proteins. In Western blots with ookinete lysates, the antisera raised against VLP-PSOP25 identified a prominent protein band at ~ 40 kDa in an identical pattern to the rPSOP25 + alum-positive control (Fig. 4b) [10]. These results showed that the VLP-PSOP25 complex induced antibodies recognizing the native PSOP25 in *P. berghei* ookinetes.

### Activities of the VLP-PSOP25 antisera inhibiting ookinete formation

**Fig. 5:**
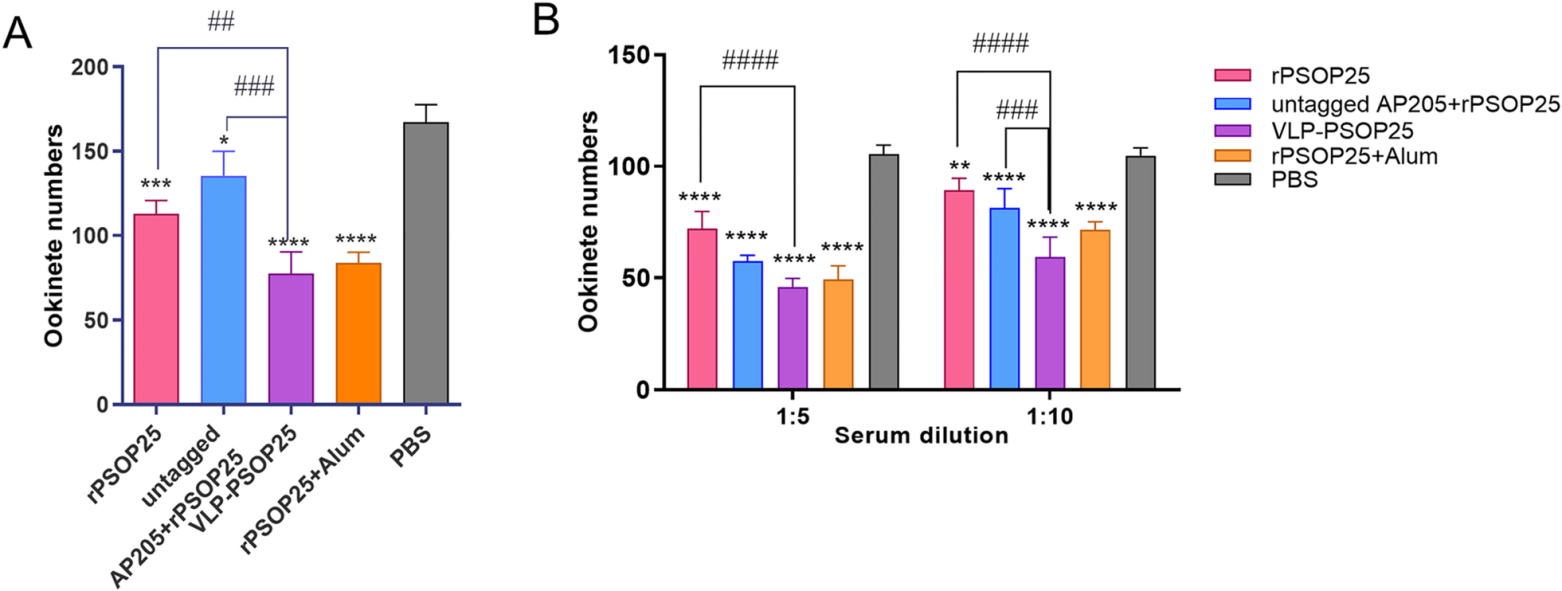
In vitro inhibition of the antisera induced by VLP-PSOP25 vaccine on ookinete formation. The inhibition activity of induced antisera was evaluated by two approaches: **a** active immunization method and **b** in vitro dilution method. Ookinetes were cultured under the same conditions, and 0.5 μl of mixed ookinete cultures was used to make smears following staining with Pbs21 mAb for ookinete counting. Data were representative of three separate experiments. Error bars were indicated as mean ± SD. *P < 0.05, **P < 0.01, ***P < 0.001, ****P < 0.0001 represent the significant difference between the respective vaccination groups and the PBS control group. ##*P* < 0.01, ###*P* < 0.001 and ####*P* < 0.0001 indicate significant differences between two immunization groups. Kruskal-Wallis test followed by Dunn’s multiple comparisons test was used for the statistical analysis

The activities of the anti-PSOP25 antibodies inhibiting ookinete formation were evaluated using an in vitro ookinete culture assay. In one study, mice were immunized as described above and infected with *P. berghei*. Infected blood was used to establish in vitro ookinete cultures. In the group of mice immunized with VLP-PSOP25, the ookinete number was reduced by 54% compared to the PBS control group (Fig. 5a). In comparison, ookinete cultures established from infected mice immunized with rPSOP25, untagged AP205 + rPSOP25, or rPSOP25 + alum showed reduced ookinete formation by 32%, 19%, and 49%, respectively, compared to the PBS group (Fig. 5a).

In another study, ookinete cultures were established from a naïve, *P. berghei*-infected mouse and supplemented with pooled immune sera raised by immunization with VLP-PSOP25, rPSOP25, untagged AP205 + rPSOP25, or rPSOP25 + alum. Compared to the PBS group, these immune sera at 1:5 dilution reduced ookinete numbers by 58%, 32%, 45%, and 54%, respectively (Fig. 5b). At 1:10, the VLP-PSOP25 antisera demonstrated a 44% reduction in ookinete number compared to the negative control group. These results provided evidence for a concentration-dependent effect of the VLP-PSOP25 antisera on ookinete formation. Moreover, the VLP-PSOP25 antisera exhibited a significantly higher inhibitory activity on ookinete formation than the rPSOP25 antisera (*P* < 0.0001, ANOVA) and untagged AP205 + rPSOP25 antisera (*P* < 0.0001, ANOVA) (Fig. 5b), whereas the VLP-PSOP25 antisera showed similar levels of inhibition of ookinete formation to the rPSOP25 + alum antisera. These results provided solid evidence about the inhibitory activity of the VLP-PSOP25 antisera on ookinete formation.

### Transmission-reducing and -blocking activities of VLP-PSOP25 antisera

**Fig. 6:**
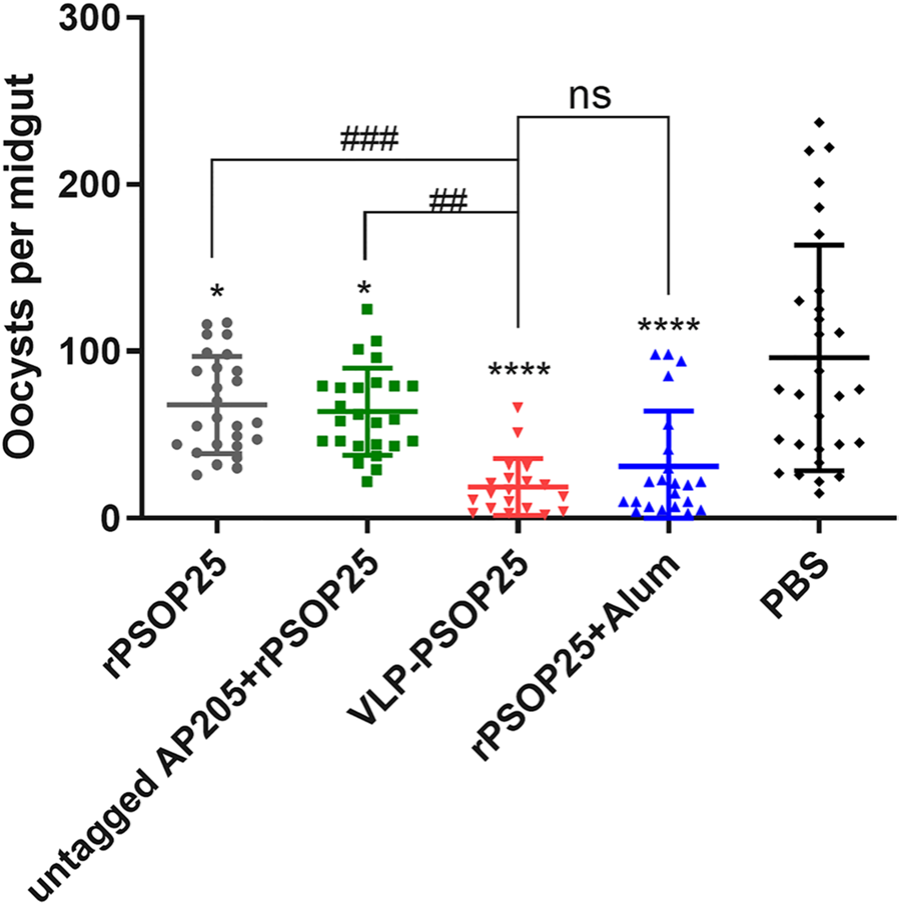
TRA of VLP-PSOP25 immunization assessed in vivo. The numbers of midgut oocysts in mosquitoes fed on mice receiving antisera from different immunization groups. The results are representative of two independent experiments. Error bars were indicated as mean ± SD. *P < 0.05 and ****P < 0.0001 indicate the significant difference between the respective vaccination group and the PBS control group. ##*P* < 0.01, ###*P* < 0.001 represent a significant difference between the two immunization groups. ns, statistically not significant. Mann-Whitney *U* test was employed for statistical analysis

To further assess the transmission reducing activity (TRA) and TBA of the VLP-PSOP25 immunization in vivo, mice infected with *P. berghei* parasites were passively transferred with different antisera through the tail vein and subjected to direct mosquito feeding assay. At 9–10 days post-feeding, 30 mosquitoes were dissected from each feeding group to determine midgut oocyst density and the prevalence of infection [9]. The mean number of oocysts per midgut in mosquitoes fed on mice receiving sera from rPSOP25 and untagged AP205 + rPSOP25 groups were reduced by 29.4% (*P* = 0.0409, Mann-Whitney *U* test; Fig. 6, Table 2) and 33.6% (*P* = 0.0162, Mann-Whitney *U* test; Fig. 6, Table 2), respectively, compared to the PBS group. The prevalence of infected mosquitoes was reduced by 10% and 13.4%, respectively (Fig. 6, Table 2). In contrast, mosquitoes fed on the mice that received the VLP-PSOP25 antisera exhibited significant reductions in both mean oocyst density (80.4%) and infection prevalence (33.4%) compared to the negative control group (*P* < 0.0001, Fig. 6, Table 2). Notably, passive transfer of antisera from mice vaccinated with rPSOP25 + alum (positive control) resulted in a slightly higher infection prevalence and oocyst densities compared to the VLP-PSOP25 group, albeit the differences were not significant (*P* = 0.7078, Fisher’s exact test; Fig. 6, Table 2). These findings suggest that the VLP display strategy elicited antisera exhibited slightly higher TRA and TBA compared to the adjuvant formulation strategy.

**Table 2:** Transmission-blocking activity of passively transferred antibodies from different immunization groups

Group	Oocyst density mean (range) (*n* = 30)	TRA^a^ (%)	Prevalence of infection mean (%) (*n* = 30)	TBA^b^ (%)
rPSOP25	67.8 (0–117)	29.4	86.7 (26/30)	10.0
Untagged AP205 + rPSOP25	63.8 (0–125)	33.6	83.3 (25/30)	13.4
VLP-PSOP25	18.8 (0–66)	80.4	63.3 (19/30)	33.4
rPSOP25 + alum	31.2 (0–98)	67.5	73.3 (22/30)	23.4
PBS	96.1 (0–237)		96.7 (29/30)	

^a^TRA was calculated as (mean oocyst density_PBS_−mean oocyst density_rPSOP25/untagged AP205 + rPSOP25/VLP-PSOP25/rPSOP25 + alum_)/mean oocyst density_PBS_ × 100%

^b^TBA was calculated as % prevalence_PBS_−% prevalence_rPSOP25/untagged AP205 + rPSOP25/VLP-PSOP25/rPSOP25 + alum_

## Discussion

Previous studies on TBVs have primarily focused on evaluating the immunogenicity and functional activity of soluble recombinant proteins [6, 36]. However, these vaccines commonly do not provide sufficient immune protection because of their inherent low immunogenicity, even in the presence of extrinsic adjuvants [37–39]. It is imperative to employ optimal vaccine delivery platforms to enhance the functional immune response of candidate antigens, thereby resulting in more efficacious vaccine formulation [40, 41]. In this study, we have successfully developed and characterized a VLP-based TBV that displays the PSOP25 antigen. The AP205-VLPs were selected as a platform due to their advantageous properties: (i) the AP205 coat protein has proved greatly receptive in tolerating foreign sequences into both the N- and C-terminus [42] and (ii) The AP205 coat protein can be produced recombinantly in *E. coli*, resulting in a high yield and low cost [26, 42]. To date, several malaria vaccine designs have been generated with the AP205 SpyTag/SpyCatcher VLP system, like Pfs48/45 [43], CSP [34], Pfs25 [44], and Pfs47 VLP vaccines [45], supporting this novel VLP strategy for increasing antibody titer and avidity. In this study, we successfully displayed the PSOP25 antigen onto the AP205 VLPs and generated homogeneous particles. Furthermore, our results demonstrated that antisera obtained from the VLP-PSOP25-immunized mice recognized the native protein PSOP25 on ookinetes, confirming that the recombinant PSOP25 protein displayed on the AP205 VLPs maintained the correct conformation to expose the specific epitopes. This work further demonstrated the compatibility of ookinete antigens with the SpyTag/SpyCatcher AP205 VLP system and the versatility of this delivery platform.

It has been reported that Spy-VLP based vaccine can enhance immunogenicity, partially because of the VLP’s ability to increase antigen display capacity, a critical factor in B cell activation [26]. To achieve optimal quantity and quality of the immune response, we incorporated two SpyTags into the VLP structure, providing a total of 360 available binding sites. The virus-like display can also disrupt B cell peripheral self-tolerance and activate anergic B cells, probably through IgD signaling. This has a remarkable positive impact on the avidity of anti-PSOP25 IgG [26, 46, 47]. Our ELISA results demonstrate that VLP-PSOP25 could give rise to antibody responses against PSOP25. In our study, a positive control group was established using a vaccine composed of soluble rPSOP25 protein and alum as an adjuvant. Alum was selected because of its widespread use in human vaccination [47, 48], since our previous studies on the immunogenicity of rPSOP25 utilized Freund's adjuvant [9, 10], which is unsuitable for human use because of its high toxicity and adverse effects [49–51]. The levels of anti-PSOP25 antibodies in the VLP-PSOP25 immunized group were comparable to those in the positive control group when examining serum samples obtained on days 28 and 38. This suggests that the VLP system is a promising alternative approach to adjuvant. The unidirectional, repetitive, and highly dense display of antigenic epitopes on the VLP surface may have also contributed to this adjuvant effect [52, 53]. Furthermore, it is noteworthy that within each group, including the VLP-PSOP25 group, three rounds of immunization produced higher levels of antibodies compared to two doses. The comparison of the avidity index between antibodies elicited by the second and third immunizations further demonstrates that the three-dose immunization scheme may enhance humoral immune responses.

Antibody subtype analysis revealed that VLP-PSOP25-vaccinated mice evoked Th1-type immune response, as evidenced by significantly higher levels of anti-PSOP25 IgG2a compared to control groups. This is desirable for vaccine efficacy since cellular immunity (Th1-like response) is associated with the level of IgG2a expression, while serum IgG1 expression level reflects humoral immunity (Th2-like response). Moreover, IgG2a exhibits a higher affinity for FcγR and possesses an enhanced capacity for complement activation compared to IgG1 [54], thereby playing a pivotal role in the clearance of intracellular parasites [55]. It has been demonstrated that the production of AP205 VLPs in *E. coli* results in encapsidation of host cell RNA, which accelerates the germinal center formation and promotes isotype switching to IgG2a by activating TLR7/8 [56].

In vitro, ookinete culture studies showed antibodies generated by vaccination with the VLP-PSOP25 reduced the number of ookinete formations. In vivo analysis of antibody functional activity showed that mosquitoes that fed on mice receiving antisera from the VLP-PSOP25 group had a reduction in oocyst density (80.4%) and a reduction in the prevalence of infected mosquitoes (33.4%). Interestingly, the VLP-PSOP25 demonstrated a slightly higher TBA or TRA to the rPSOP25 formulated in alum, which is inconsistent with ELISA data. Although the difference was not statistically significant, this suggests that functional activity in the DFA depends not only on the quantity but also on the quality of the antibodies. Leneghan et al. compared three VLP platforms displaying Pfs25 antigen, finding that AP205-VLPs exerted the best transmission-blocking effect, even though it did not elicit the highest quantity of antibodies [44]. The considerable TR activity of the antibodies produced by the VLP-PSOP25 vaccine further supports the use of the AP205-SpyTag/SpyCatcher platform for next-generation TBV development and the potential of PSOP25 as a TBV target antigen. However, one limitation of our research is that the persistence of the functional antibody response of the AP205 VLP delivery platform has not been investigated. Similar studies over a longer period of time will be required to determine the maximum duration of the VLP-based vaccine-induced antibody responses, although some studies have demonstrated that the platform display of antigens can elicit long-term antibody responses [26, 34].

Notably, the VLP-PSOP25 vaccine was not formulated with any exogenous adjuvant in this study. Previous studies have shown that some novel adjuvants can further enhance the VLP display effect and the magnitude, quality, or longevity of the elicited immunity through synergistic interplay or an additive adjuvant effect [57, 58]. For example, poly-ICLC [59], AS04 [60], Montanide ISA720 VG [43], flagellin [61], chitosan [62, 63], and three adjuvant systems (neutral liposomes/monophosphoryl lipid A/quillaja saponaria 21, squalene-in-water emulsion, and monophosphoryl lipid A) [57] may further boost immunogenicity of VLP-displayed antigen. Possibly, adjuvants formulated with the PSOP25-VLP may further improve the antigen immunogenicity and elicit more effective and persistent antigen-specific cellular and humoral immune responses.

In future studies, it will be important to optimize the VLP-based TBV to elicit higher titers of high-affinity antibodies, capable of inducing strong TRA at a lower concentration. Altogether, our study demonstrates that the non-adjuvanted VLP-based PSOP25 vaccine can induce a potent and balanced antibody response and can efficiently inhibit the development of ookinete and reduce the oocyst density in the mosquito midgut, thus representing a potential TBV candidate worthy of further investigation.

## Conclusions

In this study, we demonstrated that the VLP-based vaccine targeting the malaria antigen PSOP25 was highly immunogenic in mice, generating antigen-specific antibodies with moderate transmission-blocking effects. Notably, our findings further support the potential utility of the SpyTag/SpyCatcher AP205 VLP system in enhancing the functional efficacy of malaria TBVs.

### Supplementary Information

**Additional file 1: Figure S1.** Anti-His Western blot analysis of purified recombinant proteins. Purified **a** AP205-2*SpyTag protein, **b **PSOP25-SpyCatcher protein was detected by an anti-His antibody with a single band as Coomassie blue results shown in Fig. 1. **c** The Western blot result indicated four bands with VLP-PSOP25 component. Arrow 1 represents the VLP subunit, arrow 2 indicates the uncoupled PSOP25-SpyCatcher antigen, arrow 3 refers to a VLP subunit with a binding antigen, and arrow 4 refers to the VLP subunit with two binding antigens. The sizes of the bands are consistent with the Coomassie blue method

**Additional file 2: Figure S2.** Assessing the quality of the recombinant untagged AP205, rPSOP25 proteins. As the control group, purified untagged AP205 (**a**, **b**) and rPSOP25 (**c**, **d**) were separated by 10% SDS-PAGE gel and stained with Coomassie blue. Western blot analysis with anti-His mAb also detected the expected bands

## Data Availability

The data supporting the conclusion of this article are included within the article.
