# Supplementary figures and images for: A nanoparticle vaccine displaying the ookinete PSOP25 antigen elicits transmission-blocking antibody response against Plasmodium berghei
Source: Parasit Vectors. 2023 Nov 6;16:403. doi: 10.1186/s13071-023-06020-8 (PMC10626823; doi:10.1186/s13071-023-06020-8)

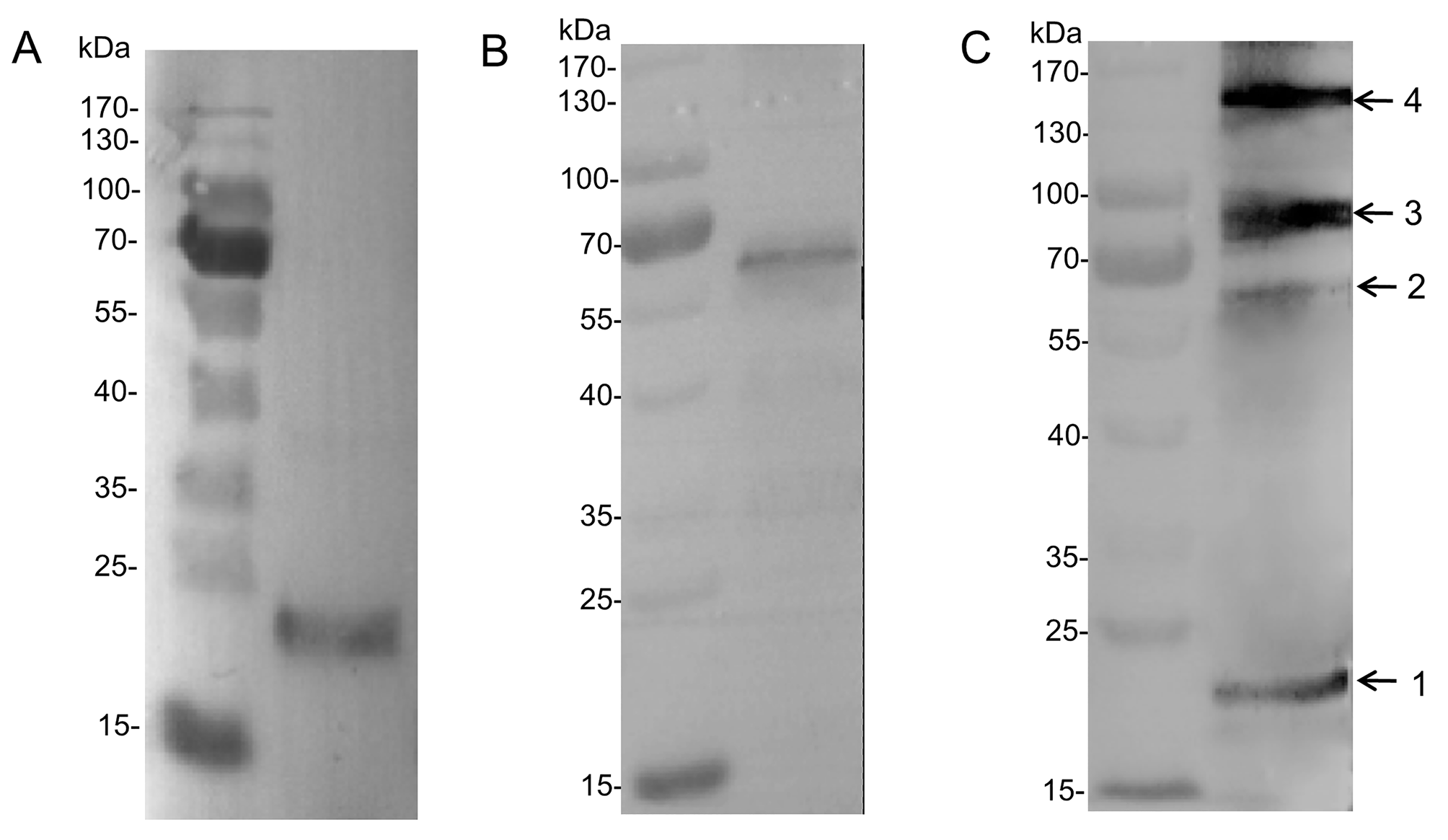

Supplement: Supplementary file 1 — Additional file 1: Figure S1. Anti-His Western blot analysis of purified recombinant proteins. Purified a AP205-2*SpyTag protein, b PSOP25-SpyCatcher protein was detected by an anti-His antibody with a single band as Coomassie blue results shown in Fig. 1. c The Western blot result indicated four bands with VLP-PSOP25 component. Arrow 1 represents the VLP subunit, arrow 2 indicates the uncoupled PSOP25-SpyCatcher antigen, arrow 3 refers to a VLP subunit with a binding antigen, and arrow 4 refers to the VLP subunit with two binding antigens. The sizes of the bands are consistent with the Coomassie blue method [file 13071_2023_6020_MOESM1_ESM.tif]

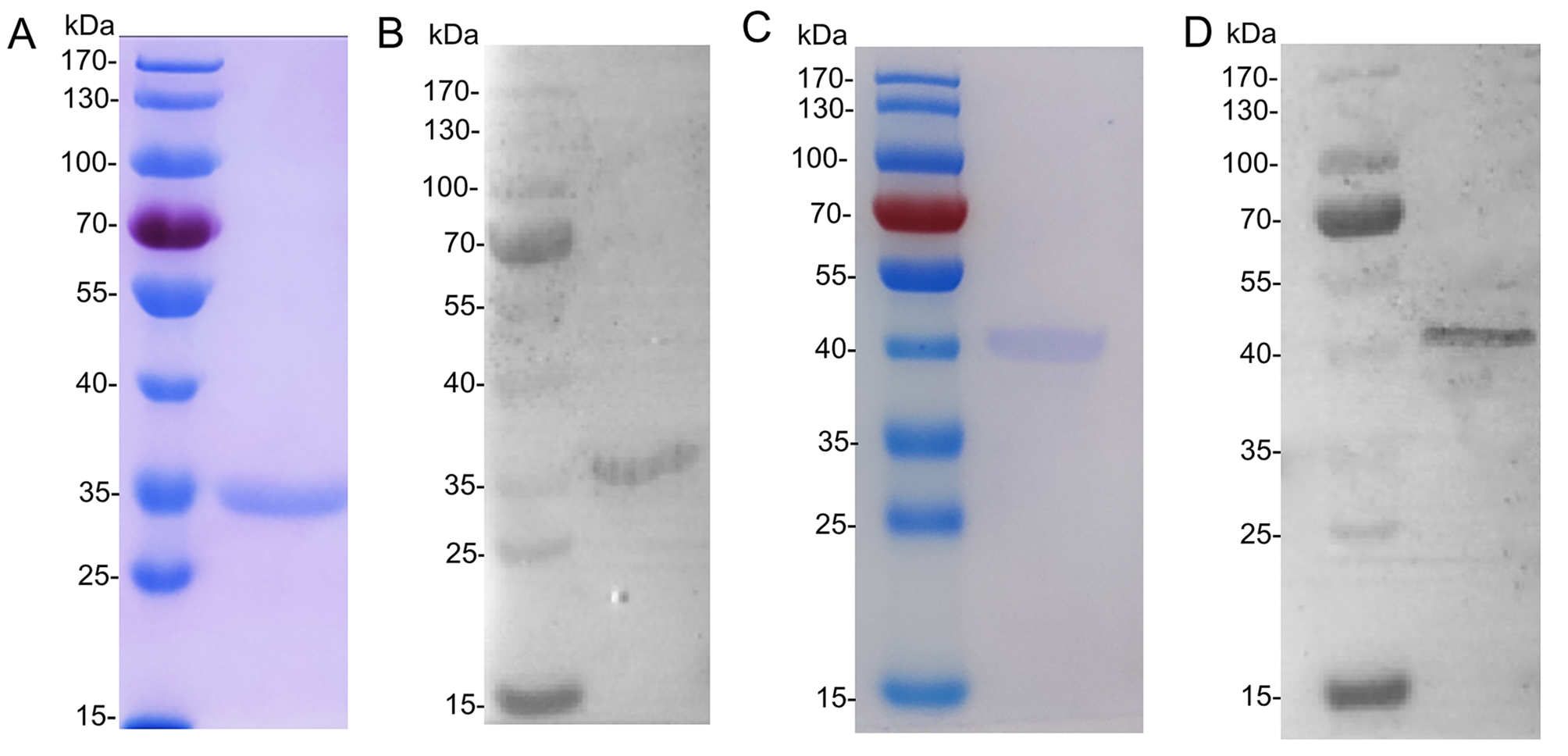

Supplement: Supplementary file 2 — Additional file 2: Figure S2. Assessing the quality of the recombinant untagged AP205, rPSOP25 proteins. As the control group, purified untagged AP205 (a, b) and rPSOP25 (c, d) were separated by 10% SDS-PAGE gel and stained with Coomassie blue. Western blot analysis with anti-His mAb also detected the expected bands [file 13071_2023_6020_MOESM2_ESM.tif]
